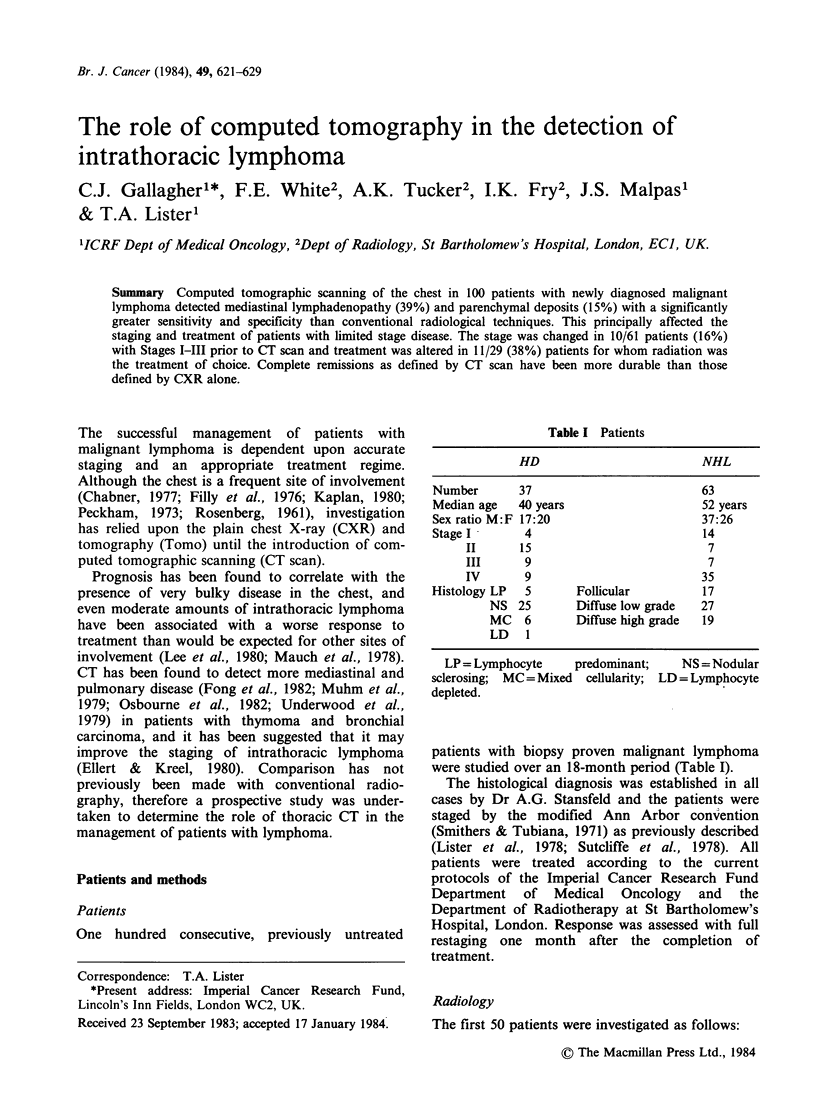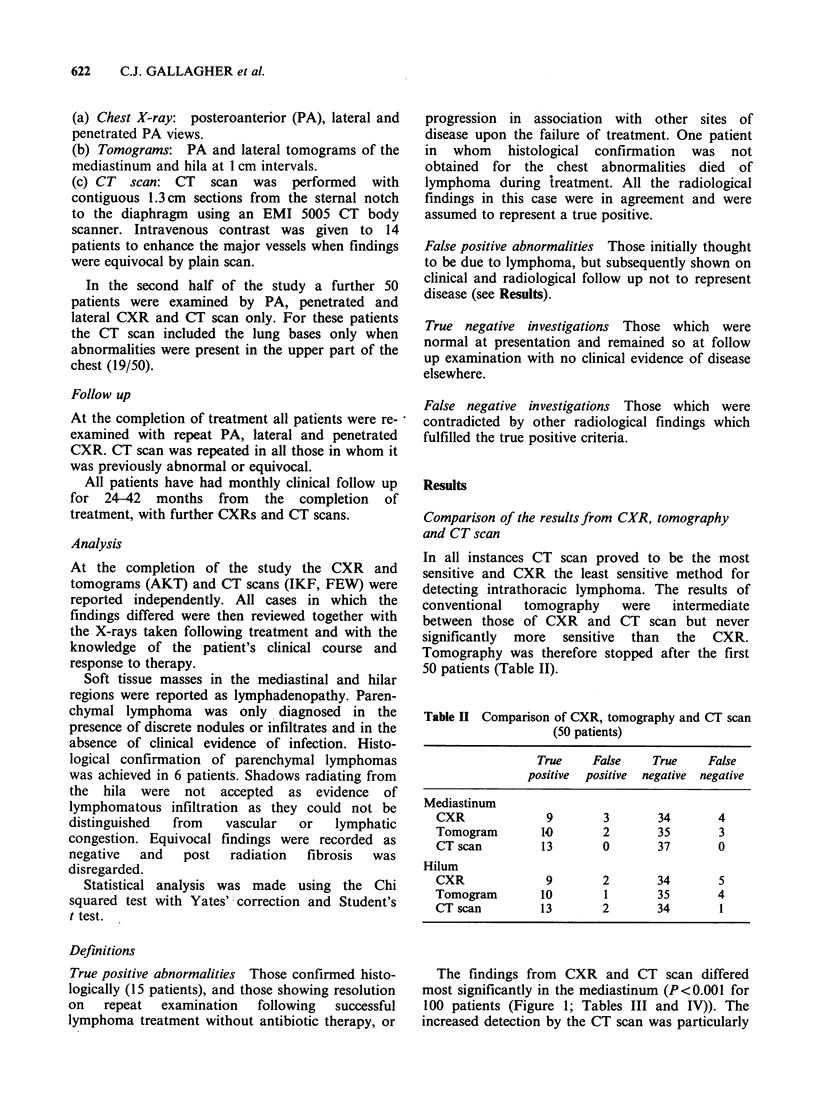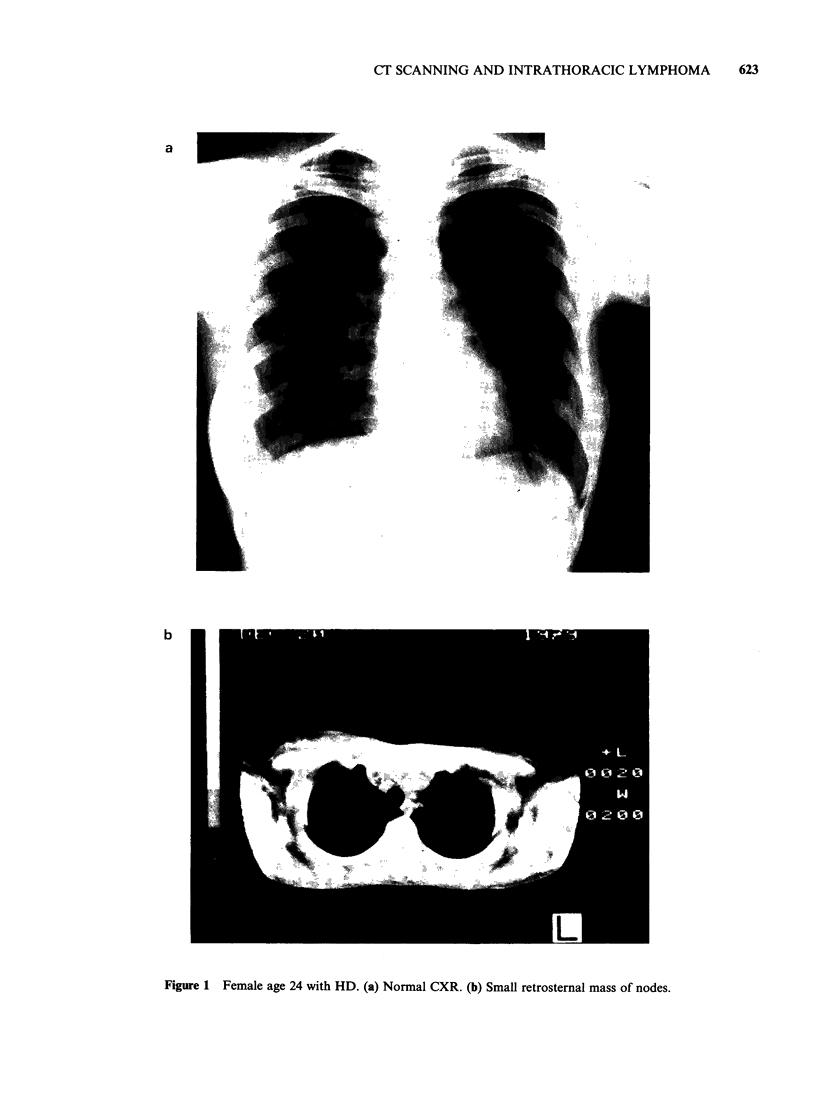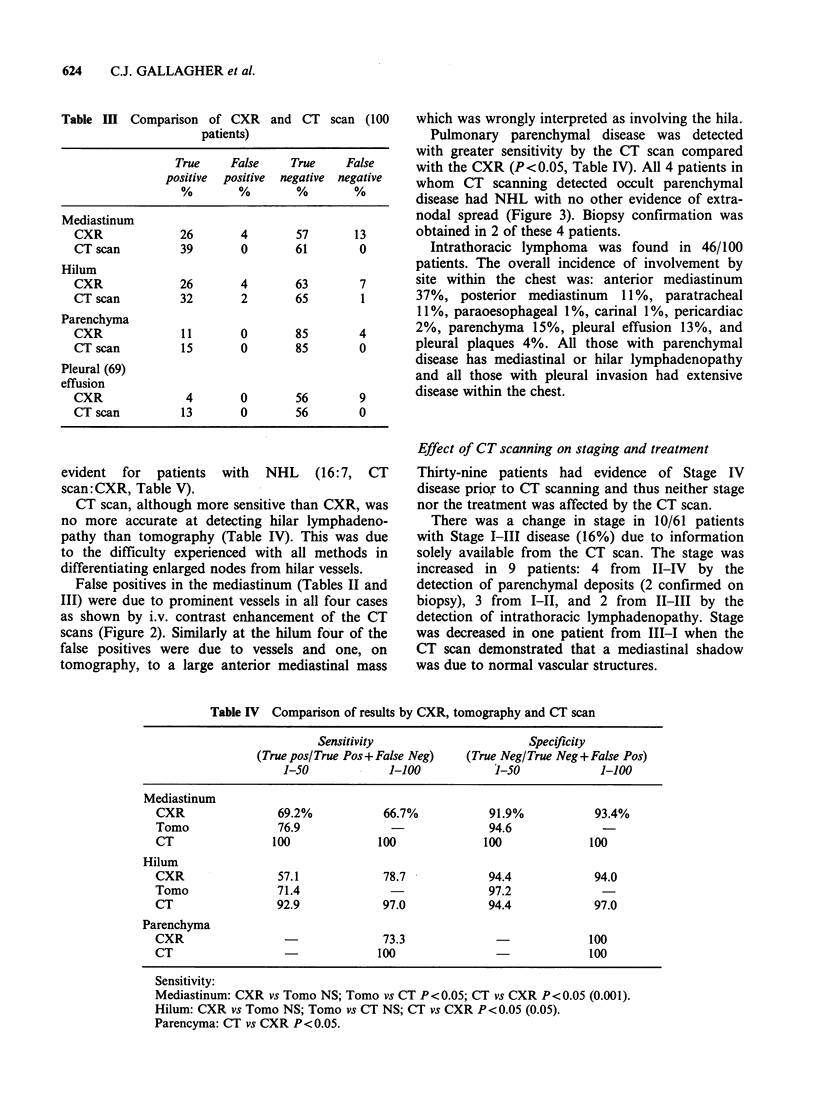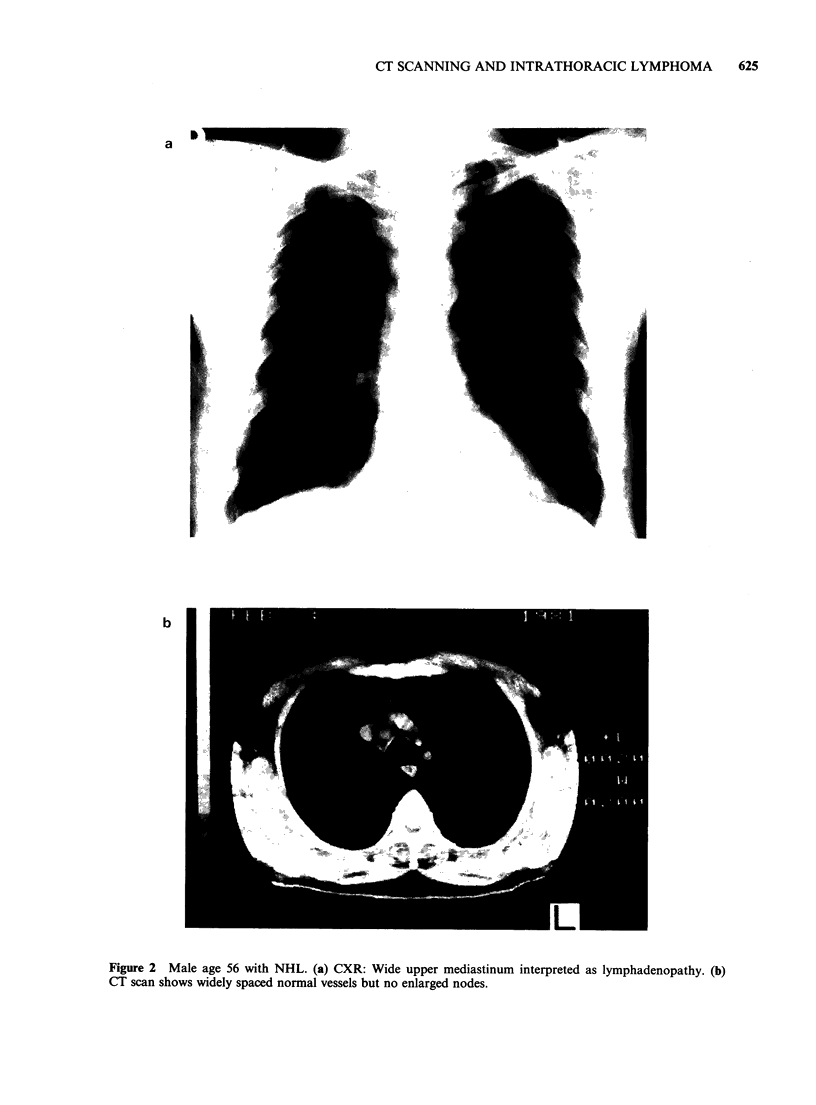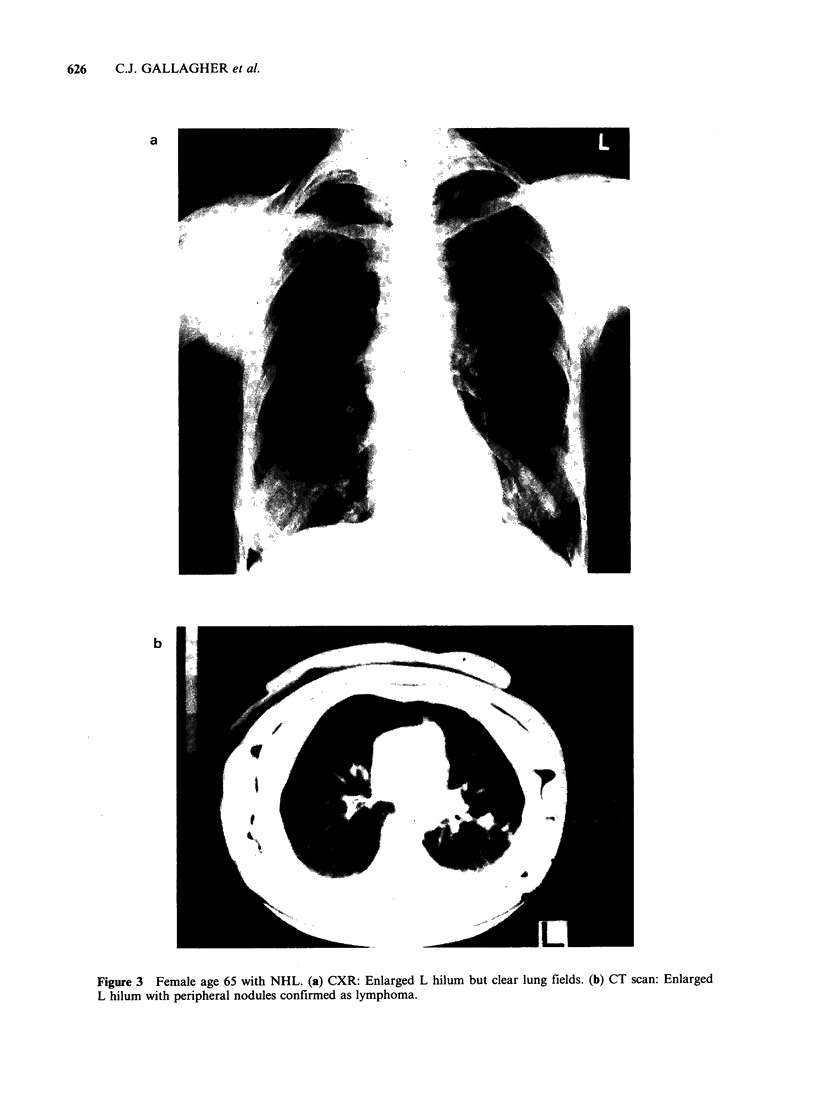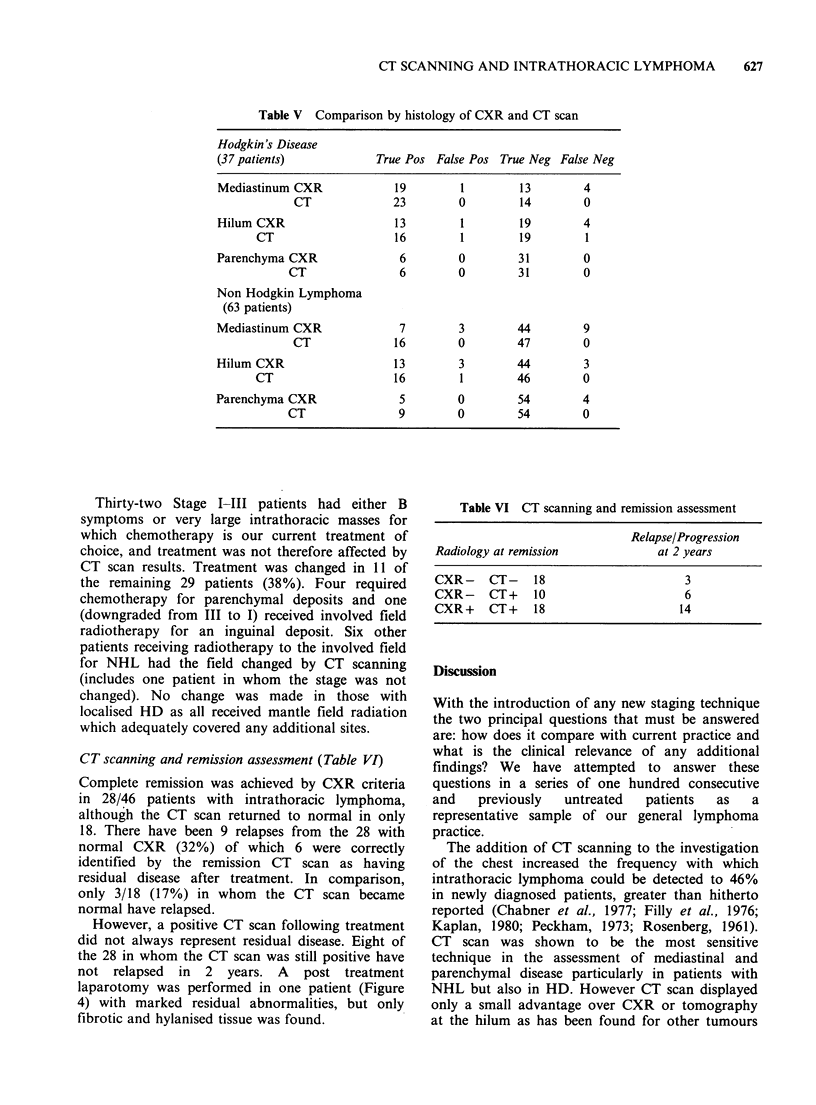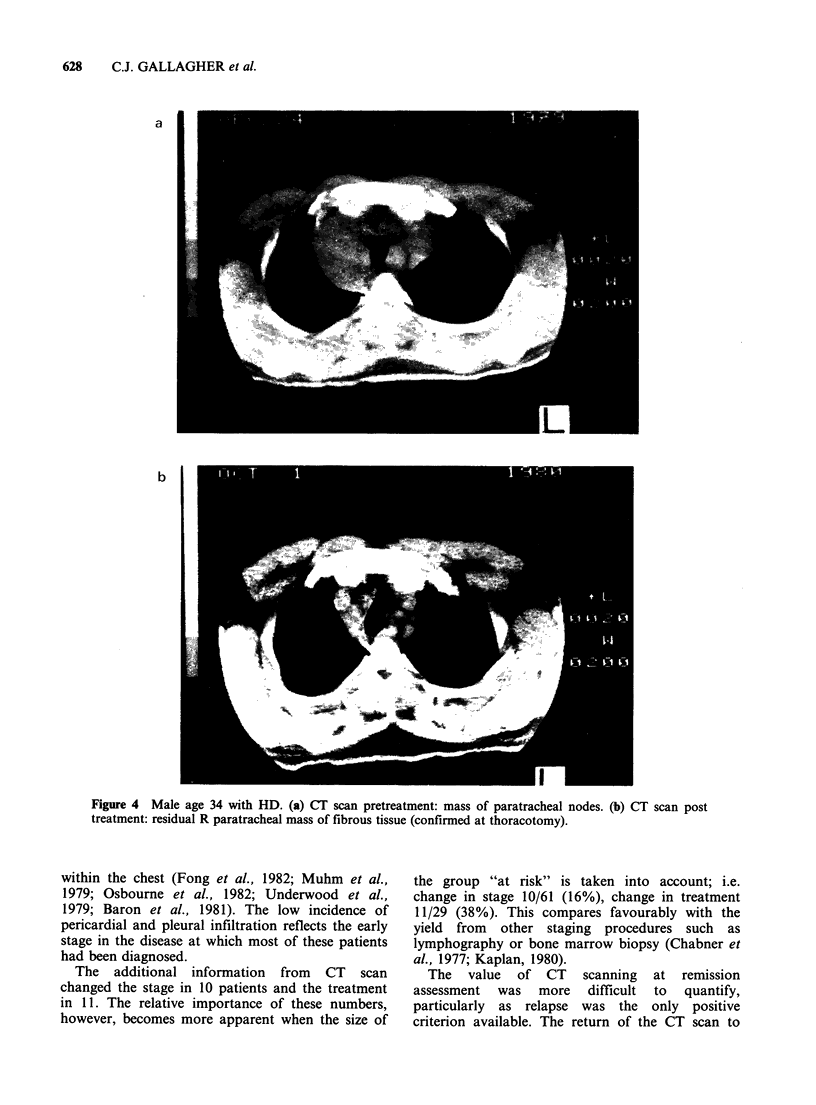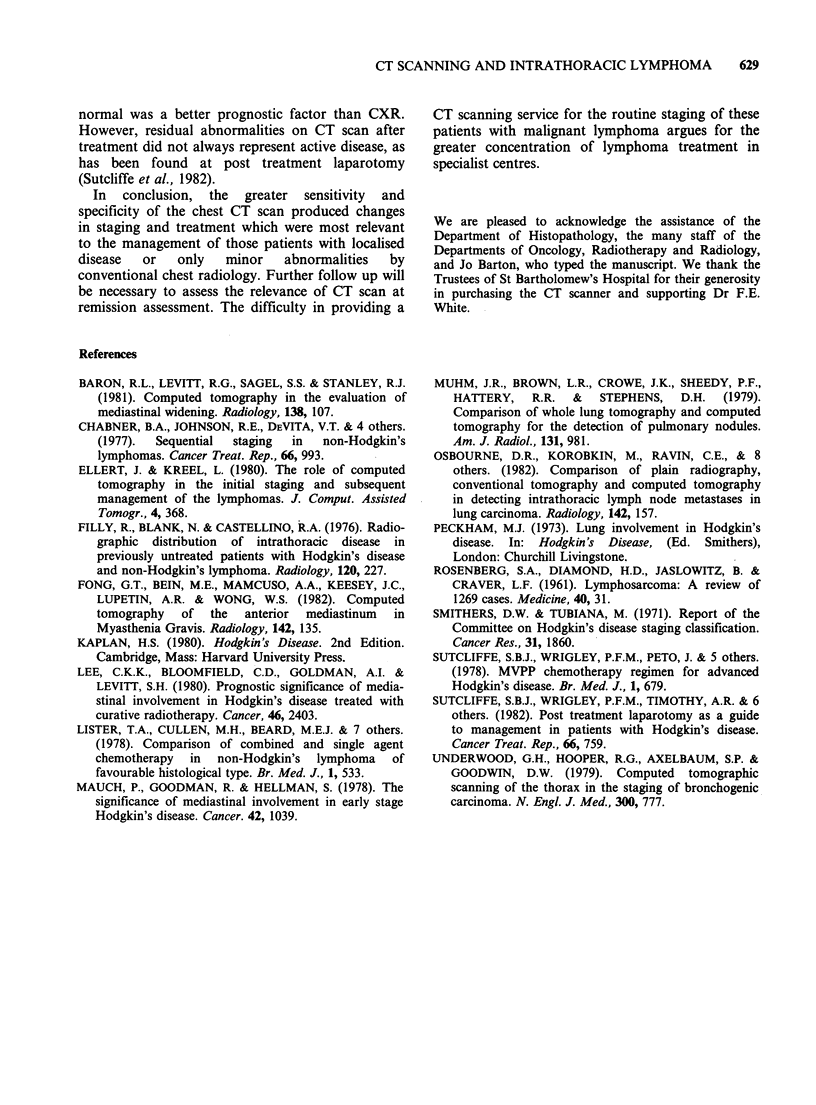# The role of computed tomography in the detection of intrathoracic lymphoma.

**DOI:** 10.1038/bjc.1984.97

**Published:** 1984-05

**Authors:** C. J. Gallagher, F. E. White, A. K. Tucker, I. K. Fry, J. S. Malpas, T. A. Lister

## Abstract

**Images:**


*Br. J. Cancer* (1984), **49**, 621–629

### The role of computed tomography in the detection of intrathoracic lymphoma

C.J. Gallagher[1*], F.E. White[2], A.K. Tucker[2], I.K. Fry[2], J.S. Malpas[1] & T.A. Lister[1]

[1]*ICRF Dept of Medical Oncology,* [2]*Dept of Radiology, St Bartholomew's Hospital, London, EC1, UK.*

**Summary** Computed tomographic scanning of the chest in 100 patients with newly diagnosed malignant lymphoma detected mediastinal lymphadenopathy (39%) and parenchymal deposits (15%) with a significantly greater sensitivity and specificity than conventional radiological techniques. This principally affected the staging and treatment of patients with limited stage disease. The stage was changed in 10/61 patients (16%) with Stages I–III prior to CT scan and treatment was altered in 11/29 (38%) patients for whom radiation was the treatment of choice. Complete remissions as defined by CT scan have been more durable than those defined by CXR alone.

The successful management of patients with malignant lymphoma is dependent upon accurate staging and an appropriate treatment regime. Although the chest is a frequent site of involvement (Chabner, 1977; Filly *et al.,* 1976; Kaplan, 1980; Peckham, 1973; Rosenberg, 1961), investigation has relied upon the plain chest X-ray (CXR) and tomography (Tomo) until the introduction of computed tomographic scanning (CT scan).

Prognosis has been found to correlate with the presence of very bulky disease in the chest, and even moderate amounts of intrathoracic lymphoma have been associated with a worse response to treatment than would be expected for other sites of involvement (Lee *et al.,* 1980; Mauch *et al.,* 1978). CT has been found to detect more mediastinal and pulmonary disease (Fong *et al.,* 1982; Muhm *et al.,* 1979; Osbourne *et al.,* 1982; Underwood *et al.,* 1979) in patients with thymoma and bronchial carcinoma, and it has been suggested that it may improve the staging of intrathoracic lymphoma (Ellert & Kreel, 1980). Comparison has not previously been made with conventional radiography, therefore a prospective study was undertaken to determine the role of thoracic CT in the management of patients with lymphoma.

### Patients and methods

### *Patients*

One hundred consecutive, previously untreated patients with biopsy proven malignant lymphoma were studied over an 18-month period (Table I).

**Table I** Patients

| | | HD | | NHL |
|---|---|---|---|---|
| Number | | 37 | | 63 |
| Median age | | 40 years | | 52 years |
| Sex ratio M:F | | 17:20 | | 37:26 |
| Stage | I | 4 | | 14 |
| | II | 15 | | 7 |
| | III | 9 | | 7 |
| | IV | 9 | | 35 |
| Histology | LP | 5 | Follicular | 17 |
| | NS | 25 | Diffuse low grade | 27 |
| | MC | 6 | Diffuse high grade | 19 |
| | LD | 1 | | |

LP = Lymphocyte predominant; NS = Nodular sclerosing; MC = Mixed cellularity; LD = Lymphocyte depleted.

The histological diagnosis was established in all cases by Dr A.G. Stansfeld and the patients were staged by the modified Ann Arbor convention (Smithers & Tubiana, 1971) as previously described (Lister *et al.,* 1978; Sutcliffe *et al.,* 1978). All patients were treated according to the current protocols of the Imperial Cancer Research Fund Department of Medical Oncology and the Department of Radiotherapy at St Bartholomew's Hospital, London. Response was assessed with full restaging one month after the completion of treatment.

### *Radiology*

The first 50 patients were investigated as follows:

Correspondence: T.A. Lister
*Present address: Imperial Cancer Research Fund, Lincoln's Inn Fields, London WC2, UK.

Received 23 September 1983; accepted 17 January 1984.

© The Macmillan Press Ltd., 1984

(a) *Chest X-ray*: posteroanterior (PA), lateral and penetrated PA views.
(b) *Tomograms*: PA and lateral tomograms of the mediastinum and hila at 1 cm intervals.
(c) *CT scan*: CT scan was performed with contiguous 1.3 cm sections from the sternal notch to the diaphragm using an EMI 5005 CT body scanner. Intravenous contrast was given to 14 patients to enhance the major vessels when findings were equivocal by plain scan.

In the second half of the study a further 50 patients were examined by PA, penetrated and lateral CXR and CT scan only. For these patients the CT scan included the lung bases only when abnormalities were present in the upper part of the chest (19/50).

*Follow up*

At the completion of treatment all patients were re-examined with repeat PA, lateral and penetrated CXR. CT scan was repeated in all those in whom it was previously abnormal or equivocal.

All patients have had monthly clinical follow up for 24–42 months from the completion of treatment, with further CXRs and CT scans.

*Analysis*

At the completion of the study the CXR and tomograms (AKT) and CT scans (IKF, FEW) were reported independently. All cases in which the findings differed were then reviewed together with the X-rays taken following treatment and with the knowledge of the patient's clinical course and response to therapy.

Soft tissue masses in the mediastinal and hilar regions were reported as lymphadenopathy. Parenchymal lymphoma was only diagnosed in the presence of discrete nodules or infiltrates and in the absence of clinical evidence of infection. Histological confirmation of parenchymal lymphomas was achieved in 6 patients. Shadows radiating from the hila were not accepted as evidence of lymphomatous infiltration as they could not be distinguished from vascular or lymphatic congestion. Equivocal findings were recorded as negative and post radiation fibrosis was disregarded.

Statistical analysis was made using the Chi squared test with Yates' correction and Student's *t* test.

*Definitions*

*True positive abnormalities* Those confirmed histologically (15 patients), and those showing resolution on repeat examination following successful lymphoma treatment without antibiotic therapy, or progression in association with other sites of disease upon the failure of treatment. One patient in whom histological confirmation was not obtained for the chest abnormalities died of lymphoma during treatment. All the radiological findings in this case were in agreement and were assumed to represent a true positive.

*False positive abnormalities* Those initially thought to be due to lymphoma, but subsequently shown on clinical and radiological follow up not to represent disease (see **Results**).

*True negative investigations* Those which were normal at presentation and remained so at follow up examination with no clinical evidence of disease elsewhere.

*False negative investigations* Those which were contradicted by other radiological findings which fulfilled the true positive criteria.

### Results

*Comparison of the results from CXR, tomography and CT scan*

In all instances CT scan proved to be the most sensitive and CXR the least sensitive method for detecting intrathoracic lymphoma. The results of conventional tomography were intermediate between those of CXR and CT scan but never significantly more sensitive than the CXR. Tomography was therefore stopped after the first 50 patients (Table II).

**Table II** Comparison of CXR, tomography and CT scan (50 patients)

| | *True positive* | *False positive* | *True negative* | *False negative* |
|---|---|---|---|---|
| Mediastinum | | | | |
| CXR | 9 | 3 | 34 | 4 |
| Tomogram | 10 | 2 | 35 | 3 |
| CT scan | 13 | 0 | 37 | 0 |
| Hilum | | | | |
| CXR | 9 | 2 | 34 | 5 |
| Tomogram | 10 | 1 | 35 | 4 |
| CT scan | 13 | 2 | 34 | 1 |

The findings from CXR and CT scan differed most significantly in the mediastinum ($P<0.001$ for 100 patients (Figure 1; Tables III and IV)). The increased detection by the CT scan was particularly

**Figure 1** Female age 24 with HD. (**a**) Normal CXR. (**b**) Small retrosternal mass of nodes.

**Table III** Comparison of CXR and CT scan (100 patients)

| | *True positive* % | *False positive* % | *True negative* % | *False negative* % |
|---|---|---|---|---|
| Mediastinum | | | | |
| CXR | 26 | 4 | 57 | 13 |
| CT scan | 39 | 0 | 61 | 0 |
| Hilum | | | | |
| CXR | 26 | 4 | 63 | 7 |
| CT scan | 32 | 2 | 65 | 1 |
| Parenchyma | | | | |
| CXR | 11 | 0 | 85 | 4 |
| CT scan | 15 | 0 | 85 | 0 |
| Pleural (69) effusion | | | | |
| CXR | 4 | 0 | 56 | 9 |
| CT scan | 13 | 0 | 56 | 0 |

evident for patients with NHL (16:7, CT scan:CXR, Table V).

CT scan, although more sensitive than CXR, was no more accurate at detecting hilar lymphadenopathy than tomography (Table IV). This was due to the difficulty experienced with all methods in differentiating enlarged nodes from hilar vessels.

False positives in the mediastinum (Tables II and III) were due to prominent vessels in all four cases as shown by i.v. contrast enhancement of the CT scans (Figure 2). Similarly at the hilum four of the false positives were due to vessels and one, on tomography, to a large anterior mediastinal mass which was wrongly interpreted as involving the hila.

Pulmonary parenchymal disease was detected with greater sensitivity by the CT scan compared with the CXR ($P<0.05$, Table IV). All 4 patients in whom CT scanning detected occult parenchymal disease had NHL with no other evidence of extranodal spread (Figure 3). Biopsy confirmation was obtained in 2 of these 4 patients.

Intrathoracic lymphoma was found in 46/100 patients. The overall incidence of involvement by site within the chest was: anterior mediastinum 37%, posterior mediastinum 11%, paratracheal 11%, paraoesophageal 1%, carinal 1%, pericardiac 2%, parenchyma 15%, pleural effusion 13%, and pleural plaques 4%. All those with parenchymal disease has mediastinal or hilar lymphadenopathy and all those with pleural invasion had extensive disease within the chest.

*Effect of CT scanning on staging and treatment*

Thirty-nine patients had evidence of Stage IV disease prior to CT scanning and thus neither stage nor the treatment was affected by the CT scan.

There was a change in stage in 10/61 patients with Stage I–III disease (16%) due to information solely available from the CT scan. The stage was increased in 9 patients: 4 from II–IV by the detection of parenchymal deposits (2 confirmed on biopsy), 3 from I–II, and 2 from II–III by the detection of intrathoracic lymphadenopathy. Stage was decreased in one patient from III–I when the CT scan demonstrated that a mediastinal shadow was due to normal vascular structures.

**Table IV** Comparison of results by CXR, tomography and CT scan

| | *Sensitivity (True pos/True Pos+False Neg)* 1-50 | *1-100* | *Specificity (True Neg/True Neg+False Pos)* 1-50 | *1-100* |
|---|---|---|---|---|
| Mediastinum | | | | |
| CXR | 69.2% | 66.7% | 91.9% | 93.4% |
| Tomo | 76.9 | — | 94.6 | — |
| CT | 100 | 100 | 100 | 100 |
| Hilum | | | | |
| CXR | 57.1 | 78.7 | 94.4 | 94.0 |
| Tomo | 71.4 | — | 97.2 | — |
| CT | 92.9 | 97.0 | 94.4 | 97.0 |
| Parenchyma | | | | |
| CXR | — | 73.3 | — | 100 |
| CT | — | 100 | — | 100 |

Sensitivity:
Mediastinum: CXR *vs* Tomo NS; Tomo *vs* CT $P<0.05$; CT *vs* CXR $P<0.05$ (0.001).
Hilum: CXR *vs* Tomo NS; Tomo *vs* CT NS; CT *vs* CXR $P<0.05$ (0.05).
Parencyma: CT *vs* CXR $P<0.05$.

**Figure 2** Male age 56 with NHL. (**a**) CXR: Wide upper mediastinum interpreted as lymphadenopathy. (**b**) CT scan shows widely spaced normal vessels but no enlarged nodes.

**Figure 3** Female age 65 with NHL. (**a**) CXR: Enlarged L hilum but clear lung fields. (**b**) CT scan: Enlarged L hilum with peripheral nodules confirmed as lymphoma.

**Table V** Comparison by histology of CXR and CT scan

| *Hodgkin's Disease (37 patients)* | *True Pos* | *False Pos* | *True Neg* | *False Neg* |
|---|---|---|---|---|
| Mediastinum CXR | 19 | 1 | 13 | 4 |
| CT | 23 | 0 | 14 | 0 |
| Hilum CXR | 13 | 1 | 19 | 4 |
| CT | 16 | 1 | 19 | 1 |
| Parenchyma CXR | 6 | 0 | 31 | 0 |
| CT | 6 | 0 | 31 | 0 |
| Non Hodgkin Lymphoma (63 patients) | | | | |
| Mediastinum CXR | 7 | 3 | 44 | 9 |
| CT | 16 | 0 | 47 | 0 |
| Hilum CXR | 13 | 3 | 44 | 3 |
| CT | 16 | 1 | 46 | 0 |
| Parenchyma CXR | 5 | 0 | 54 | 4 |
| CT | 9 | 0 | 54 | 0 |

Thirty-two Stage I–III patients had either B symptoms or very large intrathoracic masses for which chemotherapy is our current treatment of choice, and treatment was not therefore affected by CT scan results. Treatment was changed in 11 of the remaining 29 patients (38%). Four required chemotherapy for parenchymal deposits and one (downgraded from III to I) received involved field radiotherapy for an inguinal deposit. Six other patients receiving radiotherapy to the involved field for NHL had the field changed by CT scanning (includes one patient in whom the stage was not changed). No change was made in those with localised HD as all received mantle field radiation which adequately covered any additional sites.

*CT scanning and remission assessment (Table VI)*

Complete remission was achieved by CXR criteria in 28/46 patients with intrathoracic lymphoma, although the CT scan returned to normal in only 18. There have been 9 relapses from the 28 with normal CXR (32%) of which 6 were correctly identified by the remission CT scan as having residual disease after treatment. In comparison, only 3/18 (17%) in whom the CT scan became normal have relapsed.

However, a positive CT scan following treatment did not always represent residual disease. Eight of the 28 in whom the CT scan was still positive have not relapsed in 2 years. A post treatment laparotomy was performed in one patient (Figure 4) with marked residual abnormalities, but only fibrotic and hylanised tissue was found.

**Table VI** CT scanning and remission assessment

| *Radiology at remission* | | | *Relapse/Progression at 2 years* |
|---|---|---|---|
| CXR− | CT− | 18 | 3 |
| CXR− | CT+ | 10 | 6 |
| CXR+ | CT+ | 18 | 14 |

### Discussion

With the introduction of any new staging technique the two principal questions that must be answered are: how does it compare with current practice and what is the clinical relevance of any additional findings? We have attempted to answer these questions in a series of one hundred consecutive and previously untreated patients as a representative sample of our general lymphoma practice.

The addition of CT scanning to the investigation of the chest increased the frequency with which intrathoracic lymphoma could be detected to 46% in newly diagnosed patients, greater than hitherto reported (Chabner *et al.*, 1977; Filly *et al.*, 1976; Kaplan, 1980; Peckham, 1973; Rosenberg, 1961). CT scan was shown to be the most sensitive technique in the assessment of mediastinal and parenchymal disease particularly in patients with NHL but also in HD. However CT scan displayed only a small advantage over CXR or tomography at the hilum as has been found for other tumours

**Figure 4** Male age 34 with HD. (**a**) CT scan pretreatment: mass of paratracheal nodes. (**b**) CT scan post treatment: residual R paratracheal mass of fibrous tissue (confirmed at thoracotomy).

within the chest (Fong *et al.*, 1982; Muhm *et al.*, 1979; Osbourne *et al.*, 1982; Underwood *et al.*, 1979; Baron *et al.*, 1981). The low incidence of pericardial and pleural infiltration reflects the early stage in the disease at which most of these patients had been diagnosed.

The additional information from CT scan changed the stage in 10 patients and the treatment in 11. The relative importance of these numbers, however, becomes more apparent when the size of the group "at risk" is taken into account; i.e. change in stage 10/61 (16%), change in treatment 11/29 (38%). This compares favourably with the yield from other staging procedures such as lymphography or bone marrow biopsy (Chabner *et al.*, 1977; Kaplan, 1980).

The value of CT scanning at remission assessment was more difficult to quantify, particularly as relapse was the only positive criterion available. The return of the CT scan to normal was a better prognostic factor than CXR. However, residual abnormalities on CT scan after treatment did not always represent active disease, as has been found at post treatment laparotomy (Sutcliffe *et al.*, 1982).

In conclusion, the greater sensitivity and specificity of the chest CT scan produced changes in staging and treatment which were most relevant to the management of those patients with localised disease or only minor abnormalities by conventional chest radiology. Further follow up will be necessary to assess the relevance of CT scan at remission assessment. The difficulty in providing a CT scanning service for the routine staging of these patients with malignant lymphoma argues for the greater concentration of lymphoma treatment in specialist centres.

We are pleased to acknowledge the assistance of the Department of Histopathology, the many staff of the Departments of Oncology, Radiotherapy and Radiology, and Jo Barton, who typed the manuscript. We thank the Trustees of St Bartholomew's Hospital for their generosity in purchasing the CT scanner and supporting Dr F.E. White.

### References

BARON, R.L., LEVITT, R.G., SAGEL, S.S. & STANLEY, R.J. (1981). Computed tomography in the evaluation of mediastinal widening. *Radiology*, **138**, 107.

CHABNER, B.A., JOHNSON, R.E., DEVITA, V.T. & 4 others. (1977). Sequential staging in non-Hodgkin's lymphomas. *Cancer Treat. Rep.*, **66**, 993.

ELLERT, J. & KREEL, L. (1980). The role of computed tomography in the initial staging and subsequent management of the lymphomas. *J. Comput. Assisted Tomogr.*, **4**, 368.

FILLY, R., BLANK, N. & CASTELLINO, R.A. (1976). Radiographic distribution of intrathoracic disease in previously untreated patients with Hodgkin's disease and non-Hodgkin's lymphoma. *Radiology*, **120**, 227.

FONG, G.T., BEIN, M.E., MAMCUSO, A.A., KEESEY, J.C., LUPETIN, A.R. & WONG, W.S. (1982). Computed tomography of the anterior mediastinum in Myasthenia Gravis. *Radiology*, **142**, 135.

KAPLAN, H.S. (1980). *Hodgkin's Disease*. 2nd Edition. Cambridge, Mass: Harvard University Press.

LEE, C.K.K., BLOOMFIELD, C.D., GOLDMAN, A.I. & LEVITT, S.H. (1980). Prognostic significance of mediastinal involvement in Hodgkin's disease treated with curative radiotherapy. *Cancer*, **46**, 2403.

LISTER, T.A., CULLEN, M.H., BEARD, M.E.J. & 7 others. (1978). Comparison of combined and single agent chemotherapy in non-Hodgkin's lymphoma of favourable histological type. *Br. Med. J.*, **1**, 533.

MAUCH, P., GOODMAN, R. & HELLMAN, S. (1978). The significance of mediastinal involvement in early stage Hodgkin's disease. *Cancer*. **42**, 1039.

MUHM, J.R., BROWN, L.R., CROWE, J.K., SHEEDY, P.F., HATTERY, R.R. & STEPHENS, D.H. (1979). Comparison of whole lung tomography and computed tomography for the detection of pulmonary nodules. *Am. J. Radiol.*, **131**, 981.

OSBOURNE, D.R., KOROBKIN, M., RAVIN, C.E., & 8 others. (1982). Comparison of plain radiography, conventional tomography and computed tomography in detecting intrathoracic lymph node metastases in lung carcinoma. *Radiology*, **142**, 157.

PECKHAM, M.J. (1973). Lung involvement in Hodgkin's disease. In: *Hodgkin's Disease*, (Ed. Smithers), London: Churchill Livingstone.

ROSENBERG, S.A., DIAMOND, H.D., JASLOWITZ, B. & CRAVER, L.F. (1961). Lymphosarcoma: A review of 1269 cases. *Medicine*, **40**, 31.

SMITHERS, D.W. & TUBIANA, M. (1971). Report of the Committee on Hodgkin's disease staging classification. *Cancer Res.*, **31**, 1860.

SUTCLIFFE, S.B.J., WRIGLEY, P.F.M., PETO, J. & 5 others. (1978). MVPP chemotherapy regimen for advanced Hodgkin's disease. *Br. Med. J.*, **1**, 679.

SUTCLIFFE, S.B.J., WRIGLEY, P.F.M., TIMOTHY, A.R. & 6 others. (1982). Post treatment laparotomy as a guide to management in patients with Hodgkin's disease. *Cancer Treat. Rep.*, **66**, 759.

UNDERWOOD, G.H., HOOPER, R.G., AXELBAUM, S.P. & GOODWIN, D.W. (1979). Computed tomographic scanning of the thorax in the staging of bronchogenic carcinoma. *N. Engl. J. Med.*, **300**, 777.